# Dual-Metal Zeolitic Imidazolate Framework Derived Highly Ordered Hierarchical Nanoarrays on Self-Supported Carbon Fiber for Oxygen Evolution

**DOI:** 10.3390/ma15124170

**Published:** 2022-06-12

**Authors:** Xi Du, Wenjun Zhang, Maliang Zhang, Yanhong Ji, Kunmei Su, Zhenhuan Li

**Affiliations:** 1 State Key Laboratory of Separation Membranes and Membrane Processes, Tiangong University, Tianjin 300387, China; zhangwenjun970716@163.com (W.Z.); zhangmaliang@tiangong.edu.cn (M.Z.); jiyanhong@tiangong.edu.cn (Y.J.); sukunmei@tiangong.edu.cn (K.S.); 2 School of Materials Science and Engineering, Tianjin Key Laboratory of Advanced Fibers and Energy Storage, Tiangong University, Tianjin 300387, China; 3 School of Chemistry and Chemical Engineering, Tiangong University, Tianjin 300387, China

**Keywords:** zeolitic imidazolate framework, layered double hydroxide, hierarchical nanoarrays, self-supported, synergistic effect

## Abstract

The construction of highly ordered hierarchical nanoarrays is crucial for obtaining effective transition metal carbon nanomaterial electrocatalysts for oxygen evolution reaction (OER) in water splitting. Herein, we adopted a Co metal zeolitic imidazolate framework (Co-ZIF) as a precursor by ion-exchange/etching reaction with Fe(NO_3_)_3_ to obtain hierarchical N-doped Co-Fe layered double hydroxide (CoFe-LDH) in situ generated in Co-ZIF nanoarrays based on a self-supported carbon cloth (CC) substrate noted as CoFe-LDH@Co-ZIF@CC. Benefiting from the synergistic effect of these species and their highly ordered self-supported nanoarray structure, the catalytic active sites were fully exposed and highly protected in alkaline electrolyte, which significantly promoted electron transport and improved electrochemical performance. The CoFe-LDH@Co-ZIF@CC exhibited the low overpotentials of about 225 and 319 mV at 10 and 100 mA cm^−2^ with a small Tafel slope of 81.8 mV dec^−1^ recorded in a 1.0 M KOH electrolyte. In addition, it also showed a long-term durability without obvious decay after 30 h. Therefore, its remarkable OER activity demonstrates this material’s promising application in the green hydrogen energy industry.

## 1. Introduction

Renewable energy could be the dominant form of energy use in future societies, of which hydrogen energy has received increasing attention due to its high energy density, cleanliness and sustainability [1,2,3]. Electrochemical water splitting provides an attractive approach for the production of pollution-free and high-purity hydrogen energy [4]. However, this technology still faces problems, such as its low energy efficiency, high overpotential and poor stability [5,6]. This is mainly due to the inefficient oxygen evolution reaction (OER) derived from sluggish four-electron transfer, which is the major bottleneck of the water splitting [7]. Currently, commercial Ir- or Ru- based catalysts are expensive, have poor stability and are rare, which have severely hampered their widespread use. Thus, to explore and develop highly efficient and cheap non-noble-metal electrocatalysts is the key to solving the above problem.

Traditionally, carbon materials such as graphene or carbon nanotubes have been an alternative to precious metals [8]. Currently, transition-metal carbon nanomaterials, on account of their high reserves, low cost, excellent catalytic activity and environment-friendly features have become well-respected OER electrocatalyst materials, and can be used as an alternative to noble-metal catalysts [2,9]. Transition metals represented by Fe and their compounds have proven to be extremely effective OER catalysts [10]. For example, a previous study used a Fe^3+^ ion-assisted aniline polymerization strategy to embed bimetallic CoFeP nanospheres into the nitrogen-doped porous carbon framework, and excellent OER performance was recorded [11]. In other previous studies, zeolitic imidazolate framework (ZIF) materials with advantages such as a high porosity, extremely high nitrogen contents and adjustable structure have been used as a kind of important precursor for preparing N-doped transition metal carbon nanomaterial [12,13]. These materials usually have large specific surface areas, abundant active sites and various pore structures, which are particularly advantageous for electrocatalytic processes. However, many related reports have inevitably revealed a serious problem of catalyst aggregation at the micro level, resulting in buried catalytic active sites that are deeply unexposed, which is more unbeneficial for facilitating the OER. Simultaneously, most powdered catalysts need to be combined with a polymer binder for preparing the electrode, and this will significantly block the active sites and increase the interface resistance, making it difficult to meet the requirements for the high efficiency and stability of electrochemical water splitting. Thus, using ZIF as a precursor to in situ construct a self-supported electrode with a well-organized three-dimensional (3D) skeleton on a conductive fabric is an effective way to improve the electron transfer/penetration ability.

In addition, the catalytic effect of a single type of metal is limited, and the introduction of different types of metals to achieve bimetal synergy is an effective means [14]. Nevertheless, achieving a high degree of fusion between the doped metal and the precursor matrix is still a challenging task. Layered double hydroxide (LDH) has the commonly used formula of [M^2+^_1−x_ M^3+^_x_ (OH)_2_] (A^n−^)_x/n_·mH_2_O, where M^3+^ and M^2+^ represent trivalent and bivalent metal cations, and A^n−^ represents the charge-balancing anion [15]. It takes a tremendous effort to prepare LDH with the transformation of ZIF to introduce additional metal ions. For example, Nie and his coworkers reported a method to fabricate NiFe-LDH nanosheets derived from ZIF-67 in situ grown on nickel foam, which significantly improved the catalytic performance of the OER [16]. Xu and his coworkers integrated a 2D ZIF-67 nanoarray and ternary LDH CoNiAl to obtain the hierarchical ZIF-67/CoNiAl-LDH/NF electrocatalyst, which displays outstanding OER activity, having a low overpotential of 303 mV at 10 mA cm^−2^ [17]. As a result, this is an effective strategy to adjust the electronic structure distribution of the original substance, and gives full play to the synergistic effect of bimetals in order to better improve the electrocatalytic performance.

Inspired by the above considerations, we adopted Co-ZIF as a precursor by ion-exchange/etching reaction with Fe(NO_3_)_3_ to obtain the hierarchical N-doped Co-Fe LDH in situ generated in the Co-ZIF nanoarrays based on a self-supported carbon cloth (CC) substrate, which was noted as CoFe-LDH@Co-ZIF@CC. Owing to the inherent synergistic effect among these species, CoFe-LDH@Co-ZIF@CC displayed superior OER activity. When applied in a KOH electrolyte, CoFe-LDH@Co-ZIF@CC not only showed low overpotentials of 225 and 319 mV at 10 and 100 mA cm^−^^2^ current density, but also presented excellent durability. These results demonstrate this material’s promise as an application in the green hydrogen energy industry.

## 2. Experimental Section

### 2.1. Preparation of Co-ZIF@CC

Firstly, 0.58 g Co(NO_3_)_2_·6H_2_O was immersed in 40 mL aqueous solution and poured in 40 mL aqueous solution containing 1.31 g of 2-methylimidazole at room temperature. Then, a piece of 3 × 5 cm^2^ CC was chosen and acidized and immersed in the mixed solution for 8 h. After that, above CC was washed 3 times and dried at 100 °C for 8 h to achieve Co-ZIF@CC.

### 2.2. Preparation of CoFe-LDH@Co-ZIF@CC

A total of 0.18 g of Fe(NO_3_)_3_·9H_2_O was dissolved in 50 mL absolute ethanol, and one piece of Co-ZIF@CC (1 × 3 cm^2^) was soaked in the solution and slowly stirred for 1 h. Then, the sample was dried at 70 °C for 8 h to achieve CoFe-LDH@Co-ZIF@CC.

### 2.3. The Loading of the CoFe-LDH@Co-ZIF@CC and Co-ZIF@CC Catalyst

In order to calculate the quality of Co-ZIF@CC and CoFe-LDH@Co-ZIF@CC, the synthetic Co-ZIF@CC was weighed and then soaked in 1 M HCl for ultrasound for 30 min. Then, it was removed and washed with distilled water until it was neutral. After drying, it was weighed again. Since Co-ZIF@CC would react with HCl, the mass lost was the loading of Co-ZIF@CC. The mass loading of CoFe-LDH@Co-ZIF@CC was also calculated in the same way. Finally, the calculated mass loading of Co-ZIF@CC and CoFe-LDH@Co-ZIF@CC was found to be about 2 mg cm^−2^.

### 2.4. The Loading of Commercial IrO_2_@CC

As the comparison material, the loading of commercial IrO_2_@CC was required to be the same as that of CoFe-LDH@Co-ZIF@CC and Co-ZIF@CC. Thus, the commercial IrO_2_@CC catalyst was prepared by dropping IrO_2_ dispersion liquid mixture, and the quality difference of the electrode before and after dropping was 2.0 mg cm^−2^, which was the quality of IrO_2_.

## 3. Results and Discussion

**Figure 1:**
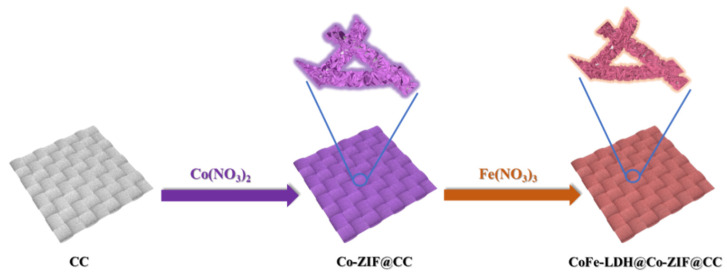
Synthesis route of hierarchical CoFe-LDH@Co-ZIF@CC electrode.

**Figure 2:**
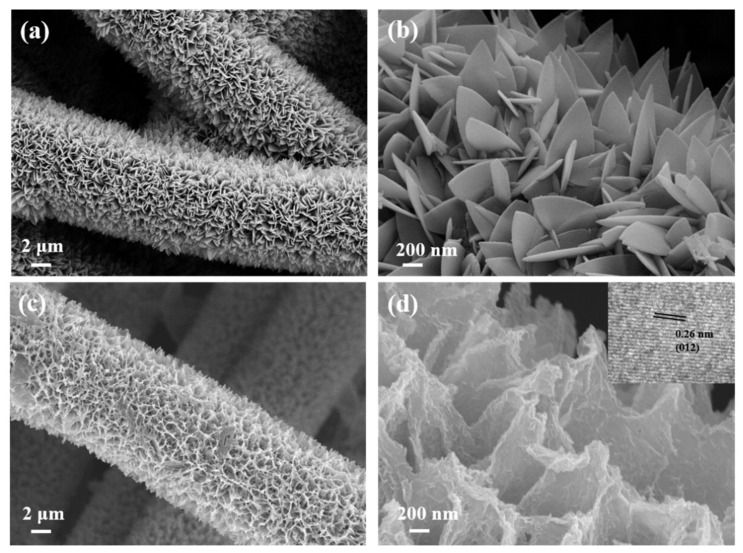
SEM images of (**a**,**b**) Co-ZIF@CC and (**c**,**d**) CoFe-LDH@Co-ZIF@CC (inset: the lattice image).

As illustrated in Figure 1, the synthesis route of CoFe-LDH@Co-ZIF clearly exhibited uniformly hierarchical nanoarrays on the self-supported CC substrate. Firstly, the acidized CC substrate was placed into a water solution mixture of Co(NO_3_)_2_·6H_2_O and 2-methylimidazole at room temperature to achieve leaf-like Co-ZIF@CC nanoarrays as a sacrificial template. Subsequently, Fe^3+^ ions were successfully introduced into the Co-ZIF@CC by ion exchange/etching process to achieve Co-Fe LDH (noted as CoFe-LDH@Co-ZIF@CC) to synergistically improve the catalytic capacity. The hierarchical nanoarrays of these materials were investigated by scanning electron microscopy (SEM). As we can see from Figure 2a,b, Co-ZIF@CC nanosheets created a fascinating hierarchical structure, with uniformly and smoothly triangular shape surfaces grown on the CC substrate. The SEM images of the CoFe-LDH@Co-ZIF@CC in Figure 2c,d show that its structural integrity was maintained well without collapse and aggregation after the ion exchange/etching process. When carefully observed, it can be seen that the overall surface arrays were quite rough with some evident “wrinkles”. It is beneficial to obtain more electroactive sites [12]. We further tested the sample for HRTME (Figure S3), and in the inset Figure 2d, the interplanar distance of 0.26 nm lattice fringe corresponds to CoFe-LDH (012).

**Figure 3:**
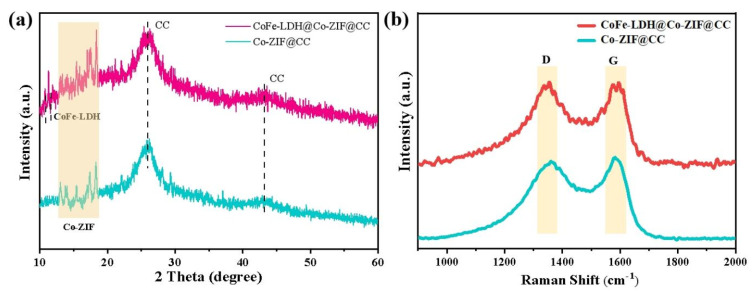
Structural characterization for CoFe-LDH@Co-ZIF@CC and Co-ZIF@CC: (**a**) XRD pattern, (**b**) Raman spectra.

The crystalline phase purity and conversion of the as-prepared CoFe-LDH@Co-ZIF@CC and Co-ZIF@CC samples were investigated by XRD. All the samples exhibited characteristic peaks at ~25° and ~43° corresponding to the interlayer spacing (002) and the plane spacing (100) of graphite carbon, respectively (Figure 3a). For Co-ZIF@CC, the strong characteristic peaks ranging from 12~18° were consistent with the reported Co-L-ZIF (Figure S4), indicating its high purity. After the ion exchange/etching process, the XRD for the CoFe-LDH@Co-ZIF@CC showed new diffraction peaks at about 11.5°, corresponding to the (003) plane of CoFe-LDH (JCPDS No. 50-0235), which implies that this formed the composite CoFe-LDH and Co-ZIF. Because the crystallinity of CoFe-LDH was weak and overlapped with the peaks of Co-ZIF and CC, only the (003) side of CoFE-LDH was observed [18]. Raman spectra of both samples exhibited two typical peaks from 1357 and 1592 cm^−1^ (Figure 3b) corresponding to the D and G bands, which indicated that there exists both defects and graphitization [19]. The ratio of *I*_D_/*I*_G_ of CoFe-LDH@Co-ZIF@CC was 1.01, larger than that of Co-ZIF@CC (0.99), indicating that there were more defects in CoFe-LDH@Co-ZIF@CC.

**Figure 4:**
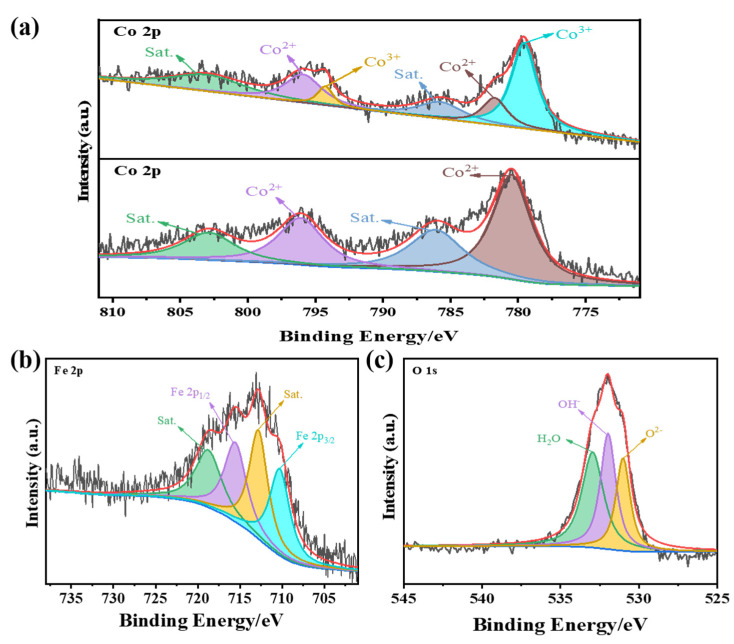
High-resolution XPS spectra of CoFe-LDH@Co-ZIF@CC: (**a**) Co 2p, (**b**) Fe 2p and (**c**) O 1s, respectively.

To further investigate the elements and valence states of the CoFe-LDH@Co-ZIF@CC catalyst, X-ray photoelectron spectroscopy (XPS) was used. As displayed in Figure S5 and Table S1, the survey spectrum of CoFe-LDH@Co-ZIF@CC demonstrated that the Co, Fe, O, C and N elements exist. The Co 2p spectrum of Co-ZIF@CC (Figure 4a) showed typical peaks at 796.1 and 780.5 eV attributed to Co^2+^ 2p_1/2_ and 2p_3/2_, along with satellite peaks at 802.8 and 786.1 eV. Unlike the single Co^2+^ oxidation state of Co-ZIF@CC, the Co 2p spectrum of CoFe-LDH@Co-ZIF@CC showed the mixed valence state of Co^2+^ (781.8 and 795.9 eV) and Co^3+^ (779.6 and 794.1 eV) [20,21,22,23,24]. This implies that the Co^2+^ cations generated by ZIF can be partially oxidized to Co^3+^ cations, thereby promoting the formation of CoFe-LDH. Upon introducing Fe, the binding energy of Co 2p for CoFe-LDH@Co-ZIF@CC shifted toward the direction of lower binding energy compared with Co-ZIF@CC, suggesting there was electronic transfer between Co and Fe. For the Fe 2p spectrum (Figure 4b), the main fitted peaks at 715.7 and 710.3 eV could be ascribed to Fe 2p_1/2_ and 2p_3/2_. These different valences are beneficial for electrocatalytic performance due to the electron transfer among them [25,26]. Moreover, the O 1s spectrum (Figure 4c) presented the characteristic peaks of an adsorbed H_2_O molecule (532.9 eV), OH^−^ (531.9 eV) and O^2−^ (531.1 eV) [27,28,29]. Thus, a small amount of iron oxide inevitably appeared in the sample during the synthesis process due to part of the Fe ion chelate with O^2−^ [30]. The C 1s spectrum (Figure S6) was divided into four peaks, ascribed to C=O (289.9 eV), C-O (286.5 eV), C-N (285.3 eV) and C-C/C=C (284.6 eV), respectively [31]. The N 1s spectrum (Figure S7) displayed its characteristic peaks at 402.9, 401.3, 400.3, 399.2 and 398.5 eV, ascribed to oxidized-N, graphitic-N, pyrrolic-N, Co-N_x_, and Fe-N_x_ species. Due to the coexistence of multi-metal, the peak position of Fe-N_x_ will have a certain deviation in the N 1s spectrum, as reported in the literature [32], but this does not deny the existence of the Fe-N_X_ locus. Doped nitrogen not only provides active sites for catalysis, but also builds metal-N bonds to allow rapid surface reconstruction in order to enhance the OER catalysis [33,34,35].

**Figure 5:**
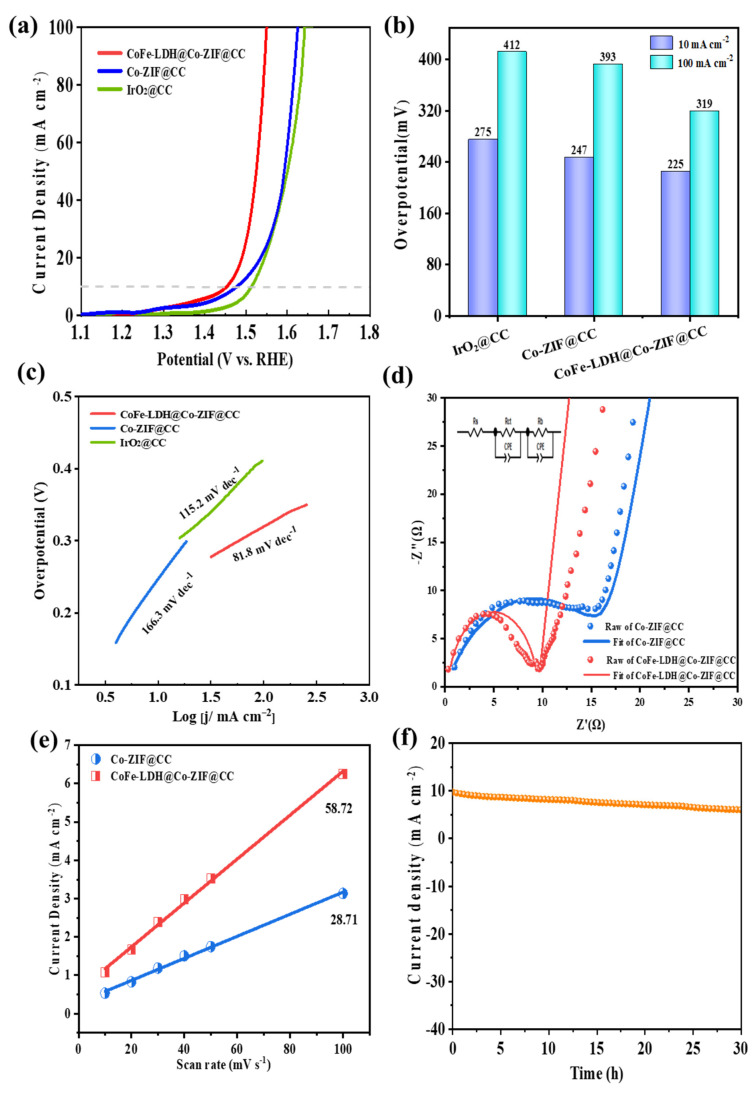
OER of as-prepared catalysts: (**a**) Polarization curves, (**b**) The overpotentials, (**c**) Tafel plots, (**d**) EIS spectra, (**e**) *C*_dl_ values and (**f**) *i-t* curve of CoFe-LDH@Co-ZIF@CC.

Encouraged by its unique structural advantages, the electrocatalytic OER activity for the CoFe-LDH@Co-ZIF@CC integrated electrode adopted the typical three-electrode system in a strong 1.0 M KOH alkaline solution. In addition, the Co-ZIF@CC and commercial IrO_2_ catalysts were also evaluated in the same condition. All the potentials were 95% IR corrected. As displayed in Figure 5a,b, at 10 and 100 mA cm^−2^ current densities, the polarization curves showed that Co-ZIF@CC can deliver the overpotentials of 247 and 393 mV, which are comparable to the commercial IrO_2_ (275 and 412 mV). Remarkably, the CoFe-LDH@Co-ZIF@CC possessed the best OER activity at 10 and 100 mA cm^−2^ with the overpotentials of 225 and 319 mV, outperforming a number of reported transition-metal carbon nanomaterial catalysts in recent years (Table S2). From Figure 5c, it can be seen that a Tafel plot based on the polarization curve of CoFe-LDH@Co-ZIF@CC exhibited excellent kinetic performance with the Tafel slope of 81.8 mV dec^−1^. It was smaller than that of Co-ZIF@CC (166.3 mV dec^−1^) and IrO_2_ (115.2 mV dec^−1^), indicating that CoFe-LDH@Co-ZIF@CC possesses fast reaction kinetics and outstanding OER catalytic activity. In addition, compared with the powder coated electrode, the self-supported CoFe-LDH@Co-ZIF@CC electrode is obviously superior to many recently reported CoFe bimetallic powder catalysts, as shown in Table S3, which further demonstrates the advantage of the self-supported electrode. EIS measurements were conducted to further study the reaction kinetics of these electrodes. The corresponding Nyquist plots are presented in Figure 5d, with an equivalent circuit shown in the inset. The semicircles of the Nyquist plot on behalf of the charge transfer resistance (R_ct_), and the smaller semicircles suggest there was a rapid charge transfer at the interface [29]. CoFe-LDH@Co-ZIF@CC exhibited much smaller R_ct_ (9.3 Ω) than Co-ZIF@CC (15.8 Ω), indicating that CoFe-LDH@Co-ZIF@CC possesses robust interfacial electron transfer kinetics and excellent electric conductivity.

To further investigate the inherent characteristics that cause such excellent OER catalytic activity, the electrochemical surface area (ECSA) for the abovementioned catalysts was measured through electrochemical double-layer capacitance (*C*_dl_). The calculated *C*_dl_ of CoFe-LDH@Co-ZIF@CC (Figure 5e and Figure S8) was as high as 58.72 mF/cm^2^, which was about 2 times larger than that of Co-ZIF@CC (28.71 mF/cm^2^). The test data were basically consistent with the fitting line. The higher *C*_dl_ value of CoFe-LDH@Co-ZIF@CC demonstrates its larger ECSA, indicating there are more active sites for OER after constructing highly ordered hierarchical nanoarrays on self-supported CC. In order to make a complete study of the electrochemical performance, the ECSA was further investigated according to ECSA = *C*_dl_/C_s_ × A. In this circumstance, C_s_ was 0.04 mFcm^−2^ in a 1.0 M KOH solution, and A indicated the geometric area of the material, which was 1 × 1 cm = 1 cm^2^. At this point, the ECSA of CoFe-LDH@Co-ZIF@CC was calculated as 1554 cm^2^, which was superior to that of Co-ZIF@CC (584 cm^2^), which is attributable to its larger reaction specific surface area and abundant active sites in this electrochemical reaction system. Combined with Tafel and EIS analysis results, we can conclude that its higher ECSA is the reason for its obvious improvement in OER activity. CoFe-LDH@Co-ZIF@CC also displayed outstanding durability after 30 h in 1.0 M KOH (Figure 5f). After the durability test, the polarization curve of CoFe-LDH@Co-ZIF@CC was close to the initial polarization curve (Figure S9). The SEM image in Figure S10 exhibits the morphological characteristics of CoFe-LDH@Co-ZIF@CC, as after the stability test the anode remained intact.

Under the guidance of the structure–activity relationship, the excellent OER activity for CoFe-LDH@Co-ZIF@CC may be due to the following factors: Firstly, the CC self-supported substrate provides a platform for CoFe-LDH@Co-ZIF to firmly root in CC without the need for additional adhesives, which ensures good electrical contact and mechanical stability. Secondly, well-organized CoFe-LDH@Co-ZIF arrays offer an electron and ion transport channel, facilitating electrolyte penetration and gas release. Thirdly, for CoFe-LDH and Co-ZIF, the synergistic effect creates abundant active sites to enhance catalytic efficiency.

## 4. Conclusions

In summary, a highly effective electrocatalyst CoFe-LDH@Co-ZIF@CC was fabricated by adopting Co-ZIF as a precursor through ion-exchange/etching reaction with Fe(NO_3_)_3_. The CoFe-LDH@Co-ZIF@CC showed excellent OER electrocatalytic activity for the overpotentials of 225 and 319 mV at 10 and 100 mA cm^−2^ current density, which is comparable to other reported transition-metal carbon nanomaterial catalysts developed in recent years. Benefiting from the synergistic effect between ZIF and LDH, this project provides an attractive approach for designing catalysts in water splitting. Considering these encouraging results already achieved, future work should be devoted to this research. However, in order to realize industrialization in the field, more in-depth research is needed to solve the following challenges: (1) The synthesis strategy needs to be continuously improved and innovated to prepare more advanced ZIF and LDH composites. (2) The composite structure needs to be further optimized to improve the electrochemical performance of the material. (3) The interaction mechanism between the composition, structure and electrochemical performance of composites should be further explored.

## Data Availability

The data presented in this study are available upon request from the corresponding author.

## Supplementary Materials

The following are available online at https://www.mdpi.com/article/10.3390/ma15124170/s1, Figure S1. SEM image of CC. Figure S2. SEM image of CoFe-LDH@Co-ZIF@CC. Figure S3. HRTEM images of CoFe-LDH@Co-ZIF@CC. Figure S4. XRD for Co-L-ZIF. Figure S5. XPS survey spectrum of CoFe-LDH@Co-ZIF@CC. Figure S6. XPS C 1s spectrum of CoFe-LDH@Co-ZIF@CC. Figure S7. XPS N 1s spectrum of CoFe-LDH@Co-ZIF@CC. Figure S8. Cyclic voltammograms of (a) CoFe-LDH@Co-ZIF@CC and (b) Co-ZIF@CC with various scan rates (10, 20, 30, 40, 50 and 100 mV/s) in 1.0 M KOH. Figure S9. Polarization curves recorded of CoFe-LDH@Co-ZIF@CC before and after the stability test. Figure S10. SEM image showing CoFe-LDH@Co-ZIF@CC as anode after stability test. Table S1. Contents of elements in CoFe-LDH@Co-ZIF@CC of XPS analysis. Table S2. Comparing OER catalytic performance for CoFe-LDH@Co-ZIF@CC with other reported transition-metal carbon nanomaterial catalysts in recent years. Table S3. OER performance of recently reported CoFe bimetallic powder catalyst. References [36,37,38,39,40,41,42,43,44,45,46,47,48,49,50] are cited in the supplementary materials.
