# Supplementary material for: Dual-Metal Zeolitic Imidazolate Framework Derived Highly Ordered Hierarchical Nanoarrays on Self-Supported Carbon Fiber for Oxygen Evolution
Source: Materials (Basel). 2022 Jun 12;15(12):4170. doi: 10.3390/ma15124170 (PMC9227379; doi:10.3390/ma15124170)
Supplement: Supplementary file 1 [file materials-15-04170-s001.zip › materials-1716415-supplementary.pdf]

Supplementary Materials

Dual-Metal Zeolitic Imidazolate Framework Derived Highly Ordered Hierarchical Nanoarrays on Self-Supported Carbon Fiber for Oxygen Evolution

Xi Du*, Wenjun Zhang, Maliang Zhang, Yanhong Ji, Kunmei Su and Zhenhuan Li*

Materials and Chemicals

CC was purchased from Tianjin Annuo New Energy Technology Co., Ltd. Nitric acid (HNO_3, 68%) and Acetone (98%) were purchased from Tianjin Sailboat Chemical Reagent Technology Co., Ltd. Absolute ethanol (99.5%), cobalt hexahydrate ($Co(NO_3)_2 \cdot 6H_2O$, 98.5%), ferric nitrate nonahydrate ($Fe(NO_3)_3 \cdot 9H_2O$, 99%) and 2-methylimidazole (99.5%) were purchased from Tianjin Kemiou Chemical Reagent Co., Ltd. Deionized water was purchased from Tianjin Yuyuan Technology Co., Ltd. All reagents were used as required without purification steps.

Material Characterization

Field emission scanning electron microscopy (FE-SEM, Gemini SEM500, Germany) was used to observe the morphologies and structures of the products. X-ray diffraction (XRD, Rigaku Smartlab 9 KW, Japan) with a Nifiltered Co Kαradiation were used to characterized material crystal structure. X-ray photoelectron spectroscopy (XPS, Thermo Scientific, ESCALAB250Xi, USA) were used to characterized surface elemental composition and chemical bonding states of the products. Raman spectroscopy (Horiba XploRA PLUS, Japan) was performed with an excitation of 532 nm laser light.

Electrochemical measurements

All the electrochemical measurements were carried out using a conventional three-electrode system on a CHI 760E (Chenhua, Shanghai) workstation at room temperature,

in which the as-prepared CoFe-LDH@Co-ZIF@CC and Co-ZIF@CC were directly used as the working electrode, graphite rod as the counter electrode and a commercial Ag/AgCl (saturated KCl) as the reference electrode. For preparing working electrode, 5.0 mg of IrO_2 was dispersed in a mixture of isopropyl alcohol (250 μL), water (250 μL), and Nafion (5 wt %, 50 μL). Then 200 μL of catalyst ink was drop dried onto a CC cloth with dimensions of 1.0×1.0 cm^2 (loading amount: 2.0 mg cm^{-2}). Linear sweep voltammetry (LSV) measurements were carried out at 5 mV s^{-1} for the polarization curves (Figure. 5a, b, c and e), previously activated by cyclic voltammetry cycle (CV) with a scan rate of 50 mV s^{-1}. Electrochemical impedance spectroscopy (EIS) measurements were performed in the frequency range of 10 mHz to 100 kHz at the amplitude of the sinusoidal voltage of 5 mV. The double-layer capacitances (C_{dl}) was tested for CV scanning at different sweep speeds (10, 20, 30, 40, 50 mV/s) in the 1.08−1.18 V vs. RHE non-faradaic potential region. All the potentials reported in our work were corrected to the reversible hydrogen electrode (RHE), according to the Nernst equation: E(RHE) =E(Ag/AgCl) +0.059 pH + 0.197, the overpotential (η) was calculated using the formula, η (V) = E (RHE) - 1.23 V. All electrochemical tests are performed in 1.0 M KOH. The cycle durability was measured by the chronoamperometric response.

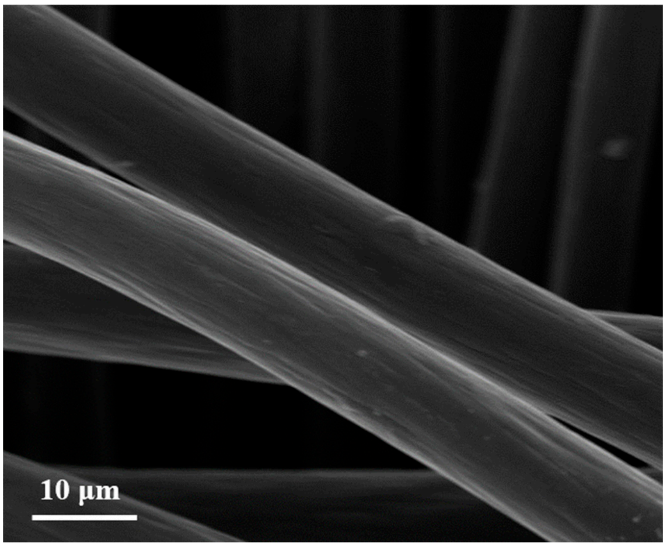

Figure S1. SEM image of CC.

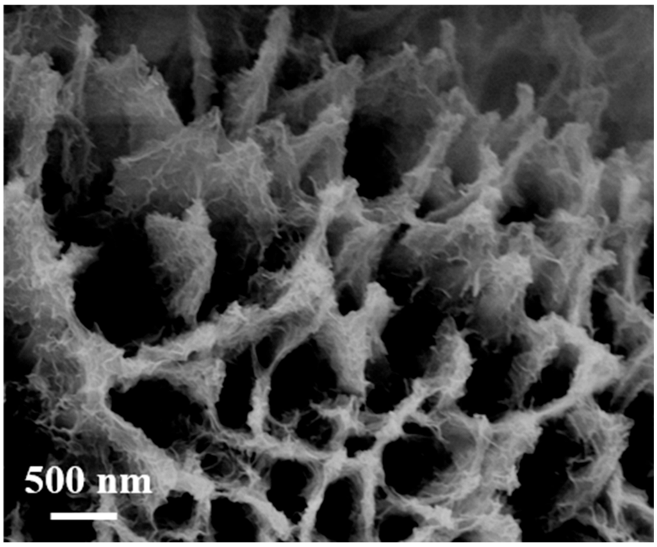

Figure S2. SEM image of CoFe-LDH@Co-ZIF@CC.

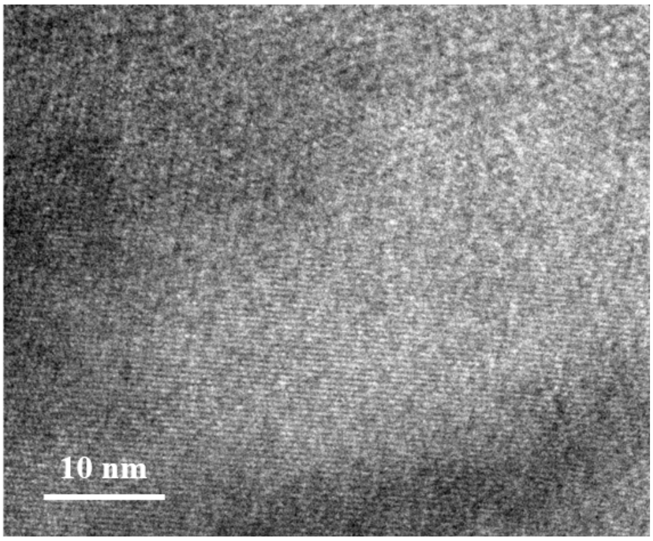

Figure S3. HRTEM images of CoFe-LDH@Co-ZIF@CC

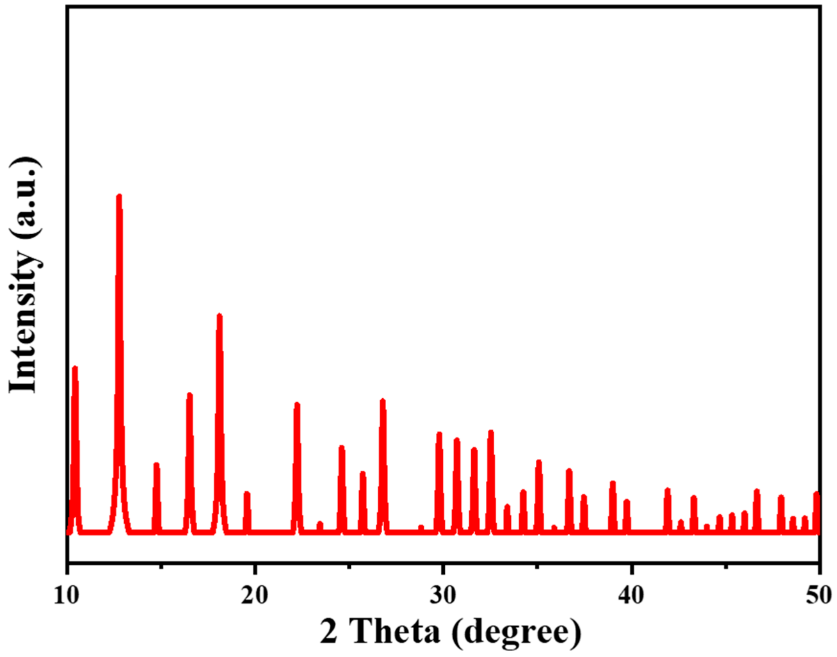

Figure S4. XRD for Co-L-ZIF.

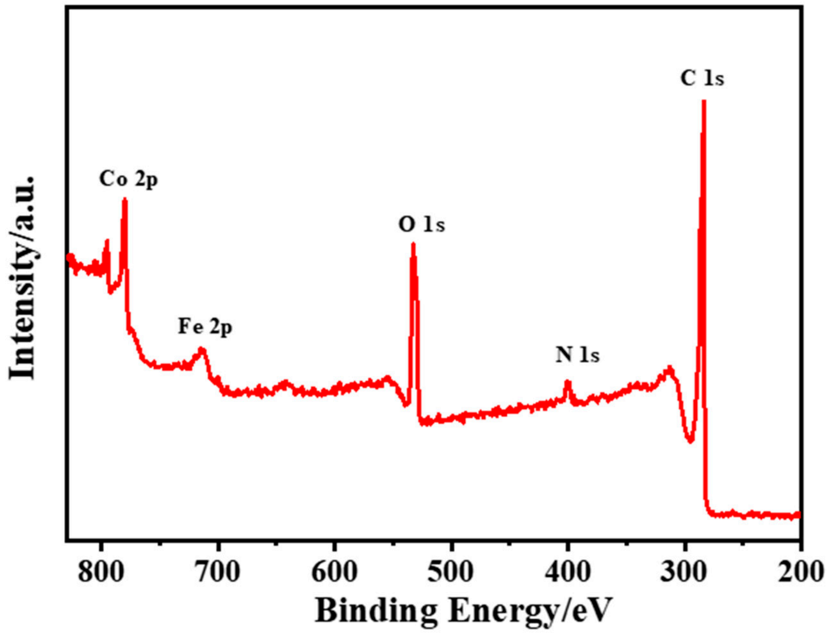

Figure S5. XPS survey spectrum of CoFe-LDH@Co-ZIF@CC.

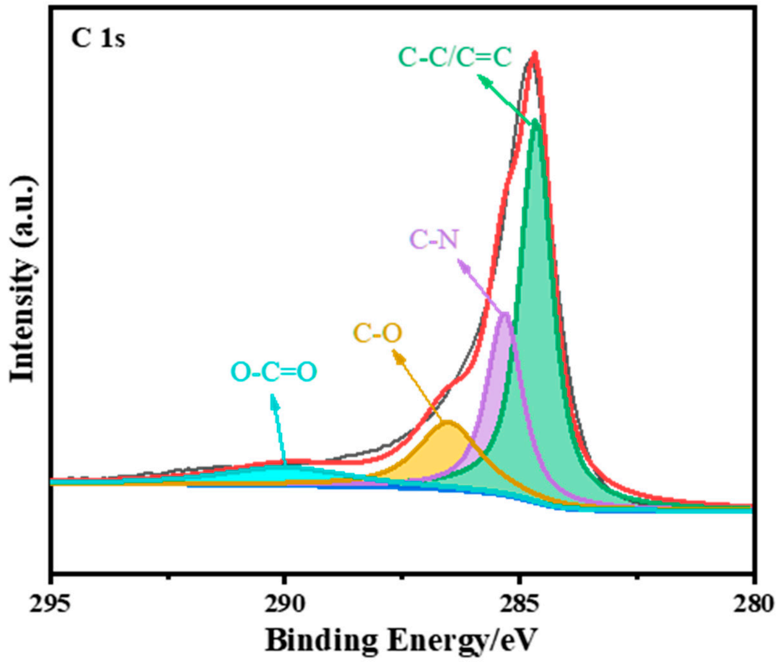

Figure S6. XPS C 1s spectrum of CoFe-LDH@Co-ZIF@CC.

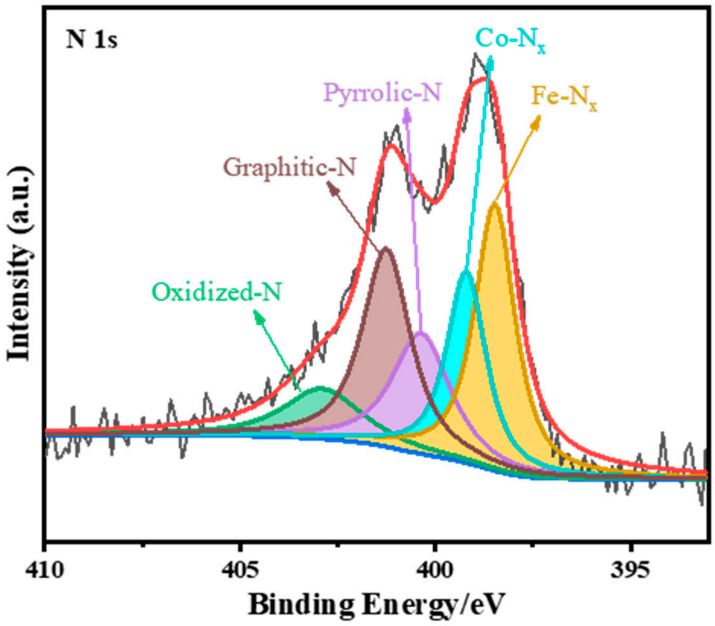

Figure S7. XPS N 1s spectrum of CoFe-LDH@Co-ZIF@CC.

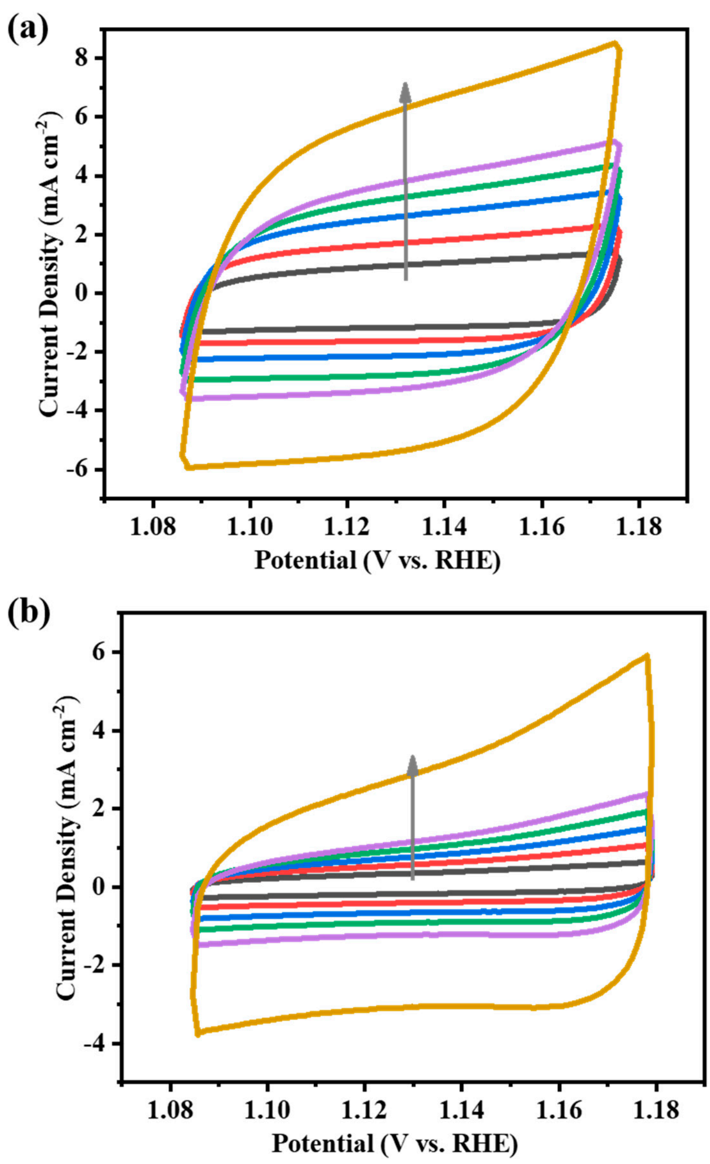

Figure S8. Cyclic voltammograms of (a) CoFe-LDH@Co-ZIF@CC and Co-ZIF@CC (b) with various scan rates (10, 20, 30, 40 , 50 and 100 mV/s) in 1.0 M KOH.

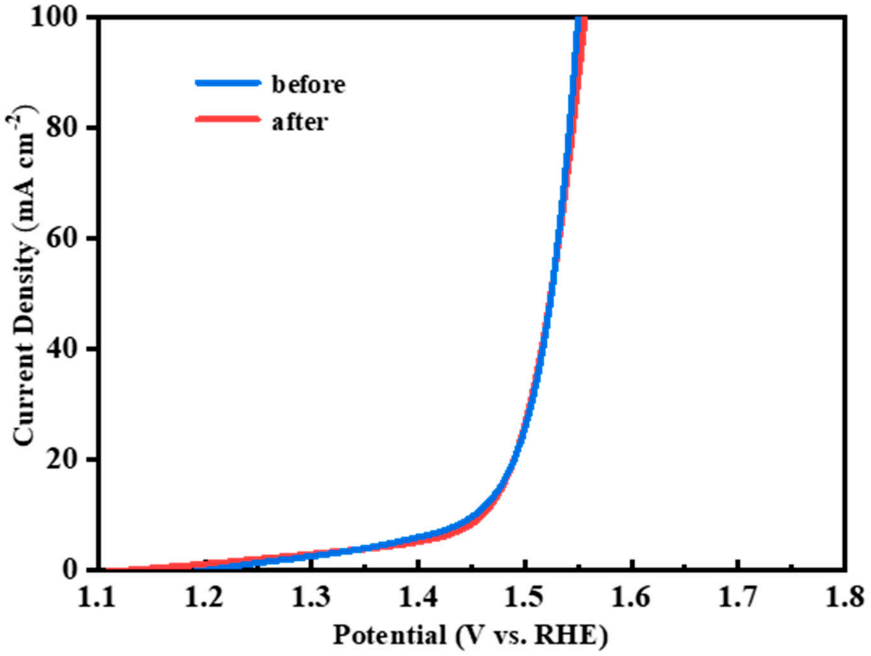

Figure S9. Polarization curves recorded of CoFe-LDH@Co-ZIF@CC before and after the stability test.

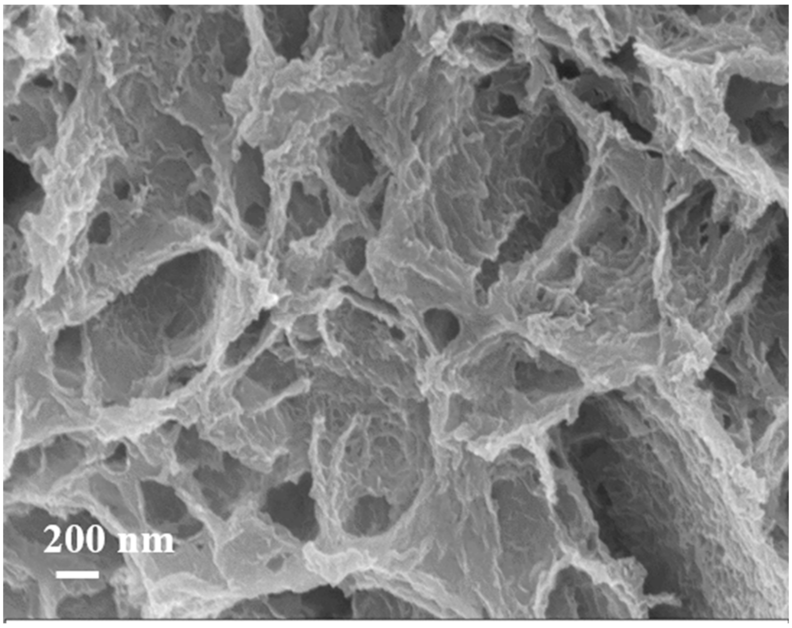

Figure S10. SEM image showing CoFe-LDH@Co-ZIF@CC as anode after stability test.

Table S1. Contents of elements in CoFe-LDH@Co-ZIF@CC of XPS analysis.

Elements	Contents
C (at%)	81.6
N (at%)	7.8
O (at%)	8.3
Co (at%)	0.5
Fe (at%)	1.6

Table S2. Comparing OER catalytic performance for CoFe-LDH@Co-ZIF@CC with other reported transition-metal carbon nanomaterial catalysts in recent years.

Catalysts	electrolyte	OER(mV)	Ref.
HEAN@NPC/CC	1.0 M KOH	263	[36]
MnFeCoNi HEA	1.0 M KOH	302	[37]
FeCo2S4@CoFe LDH	1.0 M KOH	247	[38]
CoNi@NC-NCNTs	1.0 M KOH	263	[39]
Co-Co(OH)	1.0 M KOH	264	[40]
$Ni_3ZnC_{0.7}$/NCNT	1.0 M KOH	380	[41]
$Co_{1.8}$Ni-LDH	1.0 M KOH	290	[9]
Co@NiFe-LDH	1.0 M KOH	253	[42]
NiCo-LDH/ $ZnCo_2O_4$	1.0 M KOH	260	[43]
FeCo-LDH@CoSx	1.0 M KOH	229	[44]

Table S3. OER performance of recently reported CoFe bimetallic powder catalyst.

Electrode materials	The electrolyte	OER (mV)	Ref.
$Cu_{4.76}$/CoFe LDHs	1.0M KOH	253	[45]
Fe-CoF_2-300	1.0M KOH	230	[46]
CoFe/Co_8FeS_8/CNT	1.0M KOH	290	[47]
NC_2FS-7-800	1.0M KOH	242	[48]
NCFPO-350	1.0M KOH	313	[59]
CoFe-LDH/MXene	1.0M KOH	319	[50]
